# SNiPloid: A Utility to Exploit High-Throughput SNP Data Derived from RNA-Seq in Allopolyploid Species

**DOI:** 10.1155/2013/890123

**Published:** 2013-09-12

**Authors:** Marine Peralta, Marie-Christine Combes, Alberto Cenci, Philippe Lashermes, Alexis Dereeper

**Affiliations:** UMR RPB, IRD (Institut de Recherche pour le Développement), 911 Avenue Agropolis, BP 64501, 34394 Montpellier Cedex 5, France

## Abstract

High-throughput sequencing is a common approach to discover SNP variants, especially in plant species. However, methods to analyze predicted SNPs are often optimized for diploid plant species whereas many crop species are allopolyploids and combine related but divergent subgenomes (homoeologous chromosome sets). We created a software tool, SNiPloid, that exploits and interprets putative SNPs in the context of allopolyploidy by comparing SNPs from an allopolyploid with those obtained in its modern-day diploid progenitors. SNiPloid can compare SNPs obtained from a sample to estimate the subgenome contribution to the transcriptome or SNPs obtained from two polyploid accessions to search for SNP divergence.

## 1. Introduction

The advent of high-throughput sequencing technologies is revolutionizing our ability to discover and exploit single-nucleotide polymorphisms (SNPs). Polyploidy occurs in many animals and plants but is particularly widespread in flowering plants, including many major crops. However, most methods used to discover and validate predicted SNPs are optimized for diploid species, so specific challenges related to polyploidy remain to be addressed.

Many polyploid plants including coffee (*Coffea arabica*), wheat (*Triticum durum *Desf.), cotton (*Gossypium hirsutum *L.), and peanut (*Arachis hypogaea *L.) are allopolyploids and contain two or more distinct genomes (homoeologous chromosomes) after interspecific hybridization between related diploid species and chromosome doubling. As a consequence, allopolyploid genomes hold different copies of the most of their genes and genomic merger and doubling leads to an extensive array of genomic effects, including alterations in the expression of these duplicate genes (“homoeologs”). In an allopolyploid, the chromosomes derived from different parental species do not pair at meiosis and the gene copies, “homoeoalleles” or “homoeologs,” derived from different parental species have no allelic relationships and can consequently be distinguished from true alleles. In other words, sequence variation between subgenomes coexists with allelic variation within subgenomes. Accurate identification of homoeoSNPs (i.e., polymorphisms that occurred in only one of the subgenomes) in tetraploid sequence data is a challenge due to the coassembly of homoeologs. In a co-assembly, single nucleotide differences between the two subgenomes could be confused with SNP at a single locus.

The sequencing of transcripts using high-throughput sequencing methods (RNA-Seq) can provide fresh insights into polyploid biology [1]. Typically, the reads from a given allopolyploid are aligned to a reference transcriptome. Then, if the allele sequences of the diploid progenitor species can be sampled, it is possible to infer the genome origin of the identified SNPs and to estimate the contribution of the homoeologous genes to the total transcript level.

Here we present a new tool, SNiPloid, that can tackle the many aspects involved in the analysis of SNPs in the context of allopolyploidy. Based on the coassembly of homoeologs, SNiPloid compares either putative SNPs detected from an allopolyploid to those obtained in its parental genomes, or putative SNPs derived from two allopolyploid accessions to search for polymorphism. SNiPloid web server and source code (downloadable under the CeCILL public license) are accessible at http://sniplay.cirad.fr/cgi-bin/sniploid.cgi.

## 2. Methods

### 2.1. Data Preprocessing

**Figure 1:**
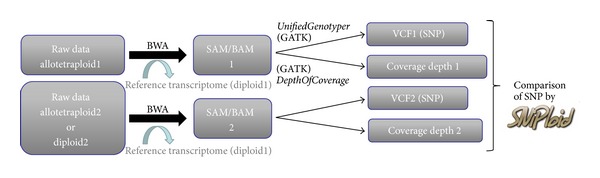
Data preprocessing. Before launching SNiPloid, each individual sample needs to be preprocessed by successively running mapping alignments and SNP calling.

Before interpreting the results of RNA-Seq data using SNiPloid, data preprocessing is required. Biologists can preprocess their data through the Galaxy public server (https://main.g2.bx.psu.edu/) as described in Figure 1.

SNiPloid assumes that short reads datasets (i.e., samples) derived from unique single genotype or distinct accessions (diploid or polyploid) are separately aligned against a single diploid transcriptome reference corresponding to one of the parental diploids using dedicated mapping software such as BWA [2], Soap [3], or Bowtie [4].

Mapping alignment is a key step in data preprocessing and mapping parameters need to be adjusted and optimized to best fit the single diploid genome used as reference. Actually, since the reference diploid transcriptome is more closely related to one of the two subgenomes in the tetraploid, it might have collateral effects on the mapping efficiency and indirectly cause biases in the interpretation of the SNP, notably when analyzing the relative homoeologous gene expression represented by the contribution of subgenomes to total gene expression. 

The SNiPloid utility uses the power of the Variant Call Format (VCF) which lists SNP variations and assigns alleles for each sequenced sample, by comparison with a reference sequence [5]. The VCF format is now widely recognized and is a standard format output of numerous SNP calling softwares. In this perspective, we suggest using the *UnifiedGenotyper* module in the GATK toolkit [6] for SNP discovery. A second type of input required by SNiPloid corresponds to a coverage depth file outputted by the *Depth Of Coverage* module of GATK. Optionally, SNP discovery and subsequent SNiPloid analysis can be improved by running the GATK *ReadBackedPhasing* utility to determine potential associations between alleles and produce phasing.

### 2.2. SNiPloid Utility

Inputs to the SNiPloid software consist of two different GATK outputs for each sample: (i) a VCF file listing putative SNPs and (ii) a coverage depth file (Figure 1). For each sample, the user can set the minimum depth coverage required to consider a position in the output statistics and the minimum minor allele frequency (MAF) required to consider the position as a variant.

**Figure 2:**
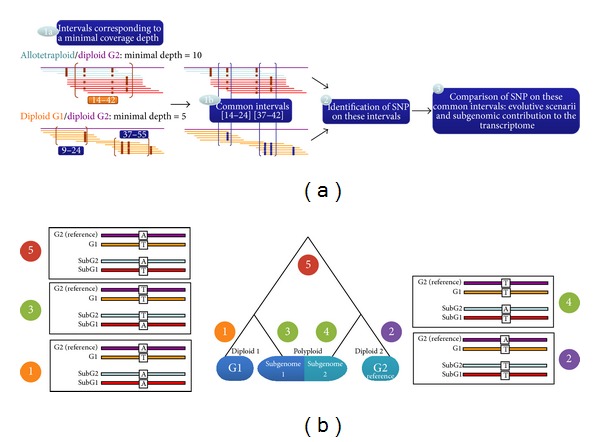
(a) SNiPloid procedure. For each reference sequence or gene of a diploid genome G2, SNiPloid extracts intervals that meet a minimal coverage depth threshold for each sample (1a) and identify overlapping intervals between samples (1b). It then extracts putative SNPs in both samples within these defined common regions (2) and compares the differences observed between samples in order to interpret the situation (3). (b) Phylogenetic contexts within a polyploidy genome and assignment of SNP categories.

SNiPloid comprises three main steps (Figure 2(a)). The first step of the utility consists in extracting regions that meet a minimum coverage depth threshold for each sample (previously set by the user) and then in identifying overlapping regions between samples. Subsequent analysis will be restricted to these regions for variant comparison. As a consequence, if putative SNPs show sufficient depth coverage in the allopolyploid but not in the diploid, or reciprocally, the position will not be processed.

In the second step also for each sample, SNiPloid extracts alleles from the VCF file for SNP positions within the defined common regions. In the third step the differences observed between samples are compared and the situation is interpreted. 

Using its main functionality (“*Polyploid versus parental diploid*”), SNiPloid offers the option to compare, interpret, and cluster SNPs. Based on the coassembly of homoeologs, SNiPloid is able to infer the SNP genome origin and distinguish interspecific SNPs and homoeoSNPs (or genome specific SNP = HSV) [7] by comparing detected SNPs in the allopolyploid to the corresponding nucleotides in both modern parental diploid genomes. SNiPloid thus classifies SNPs in different categories by hypothesizing evolution patterns as follows (Figure 2(b)).

- Patterns 1 and 2 correspond to interspecific SNPs and are assigned if an allele is specific to one of the parental genomes. The mutation occurred after the polyploidization event (e.g., diploid1 A/A, diploid2 G/G, and tetraploid G/G).
- Pattern 5 corresponds to putative homoeoSNPs because the same variation is observed in tetraploids and between parental genomes (e.g., diploid1 A/A, diploid2 G/G, and tetraploid A/G). With this pattern, SNiPloid identifies in which subgenome the homoeoallele resides by using diploid sequence alleles. In the second step, by retrieving and combining allelic depths for the reference and alternate alleles provided in the VCF format, it can estimate the subgenome contribution to the transcriptome for each homoeologous genes.
- Patterns 3 and 4 are attributed when the variation observed in the tetraploid is not identified between parental genomes (e.g., diploid1 A/A, diploid2 A/A, and tetraploid A/G). The mutation may have occurred in one of the subgenomes of the allotetraploid after the polyploidization event. With a mixture of reads originating from two subgenomes in the mapping of an allotetraploid, pattern 3 or 4 cannot be attributed without haplotype information, and a pattern “3 or 4” is assigned. In addition, SNiPloid can benefit from the phasing information included in the VCF file derived from the allotetraploid to infer the origin of an allele and distinguish between a hypothetical evolution pattern 3 or 4. Indeed, the VCF format anticipates the coding of allele phasing information (allele pairs specified by 0∣1 instead of 0/1 if phased with the previous polymorphism) in order to define haplotype blocks. Thus, if provided in the VCF, the phasing information can specify potential associations with SNP pattern 5 whose subgenome origin is known and thus distinguish between patterns 3 and 4. Basically, this process based on the haplotype makes it possible to identify putative subgenome specific SNPs.

## 3. Benefits

### 3.1. Web Application

SNiPloid is a component of the South Green Bioinformatics Platform (http://southgreen.cirad.fr) and is accessible at http://sniplay.cirad.fr/cgi-bin/sniploid.cgi as a specific utility of the SNiPlay application [8] for the analysis of allopolyploid species. 

Alternatively, SNiPloid can be downloaded as a component of the Galaxy project [9], an open-source web based computational framework that allows easy incorporation of different tools. By downloading this package, it is also possible to run the utility by command line, meaning users can manage more voluminous input datasets.

### 3.2. SNiPloid Outputs

**Figure 3:**
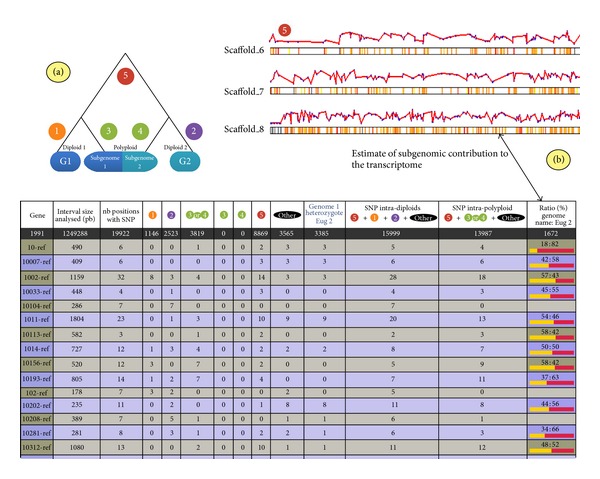
SNiPloid outputs. (a) SNiPloid produces HTML outputs showing the number of predefined SNP categories and an approximate ratio of subgenome contribution to the transcriptome for each reference sequence. (b) SNiPloid is also able to generate a graphic image that shows the overall distribution of SNP categories and of subgenome contributions along the chromosomes.

The Web application allows the export of the detailed list of classified SNPs in a tabulated format. At the end of the process, the program summarizes the analysis by counting the different SNP classes for each gene/contig of the reference dataset and by reporting the results in a dynamic sortable table (Figure 3(a)) so that users can easily classify and retrieve SNP classes of interest. For genes presenting at least one SNP class 5, an average ratio is given to obtain a global estimate of the subgenome contribution of the gene to the transcriptome. 

In addition, when the objective is to calculate general statistics or SNP frequencies along the transcriptome, the counting of SNP categories can be reported to the number of positions taken into account for the analysis, that is, positions that had met the minimum coverage depth threshold defined by the user. 

### 3.3. Comparison of Two Samples

Basically, the second option “*Polyploid versus polyploid*” of the application makes it possible to quickly distinguish and count specific and shared SNPs between two samples. The comparison can be made at three different levels: either between two samples originating from a single polyploid accession, or between two polyploid accessions, or more generally between two species. By using this functionality, new original approaches based on differential SNP can emerge for the study of the genome structure of polyploids or of the subgenome contribution to gene expression.

### 3.4. SNiPloid Map Viewer

Finally, SNiPloid includes a viewer that allows a graphical overview of the distribution of SNP categories and of subgenome contributions along the chromosomes (Figure 3(b)).

This functionality can only be applied on species for which a complete and fully annotated reference genome sequence is available and requires a structural genome annotation in General Feature Format (GFF) format as additional input, supplying the viewer program with the coordinates of gene models used as reference on the genome. The aim is to rapidly localize potential highly bias-expressed regions, introgressed genes, or homogenized regions within the genome.

### 3.5. Examples of Use Case

A whole transcriptome analysis was conducted on the allotetraploid *Coffea arabica* by using the SNiPloid software for the analysis of the contribution of subgenomes to the transcriptome [10]. This study enabled to characterize genome-wide homoeologous expression gene expression in *C. arabica*, a recent allopolyploid combining two subgenomes that derive from two closely related diploid species: *C. canephora *and *C. eugenioides*. Different samples of *C. arabica* obtained at contrasted temperatures and one *C. eugenioides* sample were mapped against the *C. canephora* reference transcriptome, analyzed for SNP discovery, before being compared with SNiPloid in order to estimate homoeologous gene expression and to highlight potential variation between growing conditions. Additionally, by mapping reads against the *C. eugenioides *transcriptome instead of *C. canephora*, this study showed that the relative homoeologous gene expression is slightly biased in favour of the genome used as reference, as anticipated above.

Sampled from this study, an example of datasets is provided by the SNiPloid Web server to familiarize users with the correct input and expected results.

### 3.6. Performance and Limitations

The main functionality of SNiPloid is dedicated to RNA-Seq data and to polyploid species for which a diploid transcriptome reference is available for at least one of the parents.

One limitation of the use of RNA-Seq for SNP detection and subsequent interpretation is that the transcript sequences represent only the expressed part of the genome and that the sequencing depth varies considerably across the genome due to the different gene expression levels. Thus, only SNPs in well-expressed genes can be detected and allele or homoeolog expression bias could make the detection of certain SNP difficult due to their low frequency in the transcriptome. However, NGS technologies and the use of appropriate read cutoffs allow to detect and interpret SNPs for a large number of genes distributed across the genome.

Theoretically, even though the allele expression quantification would not be performed, a genome wide analysis would be also possible on genomic data. However from a technical point of view, whole genome analysis would be difficult to perform through our Web server, since it requires uploading VCF and depths file inputs that would be sizeable and should be computed by command line after having downloaded the SNiPloid package or through Galaxy.

In terms of performance, in our practical experience two RNA-Seq samples derived from a polyploid and a diploid species first mapped against a complete reference transcriptome and then generating 600 000 putative SNPs each can be successfully compared by SNiPloid Web server in less than five minutes.

### 3.7. Comparison with Other SNP Bioinformatics Tools

Even though numerous SNP bioinformatics tools or pipelines exist for SNP calling (GATK [6], VarScan [11], WEP [12], and MiST [13]) or SNP annotation (SNPEff [14]) at a whole genome scale, only a few software packages allow to automatically categorize and interpret putative SNPs from polyploid species. 

An example of pipeline reported by Hand et al. [15] predicts the subgenome-specific origin of SNPs using a phylogenetic approach based on comparison with orthologous sequences from predicted progenitor species. More recently a new pipeline called PolyCat [16] has been developed for mapping and categorizing NGS reads produced from allopolyploid organisms. Having the same aim as SNiPloid, the approach is a little bit different. PolyCat uses reads from diploids to generate preindexed homoeoSNPs that will be then used to assign reads from tetraploids to a subgenome. The subgenome attribution is made at the read level whereas SNiPloid manages the subgenome attribution by considering SNPs position by position, counting homoeoSNPs for each transcript of a whole transcriptome analysis. 

This approach is relevant and more advanced but can appear slightly more fastidious to operate. The main advantage of SNiPloid is its ease to be applied since it does not require preliminary work to establish homoeoSNPs database that can be time-consuming, and offers to non-bioinformaticians a ready-to-use Web server allowing to rapidly obtain subgenome attribution thanks to a “one click” analysis.

In addition, our approach seems to be more appropriate for allopolyploid species for which the polyploidization event is relatively recent in the evolution such as Coffea or Spartina.

## 4. Conclusions

To our knowledge, SNiPloid is the first Web tool dedicated and optimized for the SNP analysis of RNA-Seq data obtained from an allopolyploid species. By exploiting the well-organized information stored in the standard VCF format, SNiPloid helps to interpret putative SNPs detected in a whole transcriptome by a comprehensive SNP categorization. SNiPloid is appropriate for allotetraploids and opens new prospects for investigating allopolyploid genome structure or expression.
